# Study on the Pressure Bearing Capability of Folded Multi-Port Flat Tube

**DOI:** 10.3390/ma12223744

**Published:** 2019-11-13

**Authors:** Ding Tang, Xiaole Chen, Leilei Zhao, Tianxia Zou, Huamiao Wang, Dayong Li, Yinghong Peng, Peidong Wu

**Affiliations:** 1 School of Mechanical Engineering, Shanghai Jiao Tong University, Shanghai 200240, China; tangding@sjtu.edu.cn (D.T.); leileizhao233@163.com (L.Z.); txzou1984@163.com (T.Z.); dyli@sjtu.edu.cn (D.L.); yhpeng@sjtu.edu.cn (Y.P.); 2 State Key Laboratory of Metal Extrusion and Forging Equipment Technology, China National Heavy Machinery Research Institute Co., Ltd., Xi’an 710032, China; xiaolechan@163.com; 3 Department of Mechanical Engineering, McMaster University, Hamilton, ON L4S 4L7, Canada; peidong@mcmaster.ca

**Keywords:** folding, multi-port flat tube, pressure bearing, bulging, bending

## Abstract

The pressure bearing capability of a folded multi-port flat tube (MPFT), which has the advantage of retaining the corrosion property of corrosion resistant materials, was investigated in this study with both a burst pressure test and finite element simulation. Results show that the folded tube’s failure is mainly caused by the breaking of the inner ribs. Instead of detecting inner pressure, the bulging ratio, which is supposed to be small under service pressure, rises rapidly before failure. Therefore, it is suggested to use bulging ratio to visibly determine the working status of folded MPFTs. Based on FE simulations, the pressure bearing capability of the folded MPFT was improved by optimizing the relevant folding parameters. In addition, the influence of in-plane bending was also investigated. It is found that the folded MPFTs can still retain most of the pressure bearing capability after in-plane bending.

## 1. Introduction

Aluminum (Al) flat section tubes are widely used in Heating, Ventilation, Air Conditioning & Refrigerating (HVAC&R) application [1,2]. Multi-port flat tubes (MPFTs) are mostly processed via extrusion, which divides an Al billet into two materials and flows through a porthole die, and rejoins and seam welds them together in the welding chamber. Since the service conditions usually involve inner pressure, the pressure bearing capability is a crucial property of an MPFT [3,4,5,6,7,8]. A high press force is required to ensure the quality of the seam welding zone of an extruded MPFT, which in turn significantly increases the manufacturing cost [9,10]. In addition, post-treatment on both the inner and outer surfaces of an extruded MPFT is not effective to improve corrosion resistance [3]. The limited service life of an extruded MPFT is still the main constraint for its wide application.

**Figure 1:**
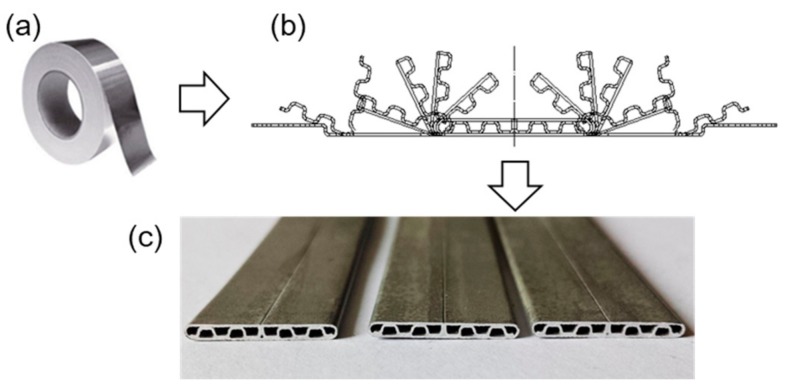
Fabricating process of a folded MPFT Multi-port flat tubes (MPFTs): (**a**) aluminum clad strip, (**b**) folding process, and (**c**) seam welded folded tube after brazing.

Alternatively, an MPFT can be produced in terms of folding by using roll forming technology (Figure 1), where an Al strip with cladded layers, to prevent corrosion, is folded through multiple passes [9]. An airtight MPFT is achieved with the post treatment of brazing. Since the cladding layers on both sides of the Al strip are corrosion resistant, the final MPFT can serve well under extreme environments, such as high humidity, high basicity, etc. Similarly, the pressure bearing capability is critical for a folded MPFT, which has barely been investigated. Moreover, some folded MPFTs might need further bending to meet the geometry requirement when assembling the HVAC&R devices [11,12]. Therefore, careful study on the mechanical properties of folded MPFTs and possible influencing factors is highly demanded.

In this work, air-tight folded MPFTs with eight ports were prepared. Bursting pressure tests were carried out to evaluate the pressure bearing capability of the tubes. In order to study the influence of in-plane bending, folded MPFTs with in-plane bending were prepared and tested. Together with finite element (FE) simulations, the failure mode of the tube under the burst pressure test was analyzed. Key factors that influenced the tube’s pressure bearing capacity were discussed. Moreover, suggestions on improving the performance of folded MPFTs were given. The relevant findings can be widely applied to other folded tubes, which are used more and more in industry.

## 2. Experiments

### 2.1. Material

**Figure 2:**
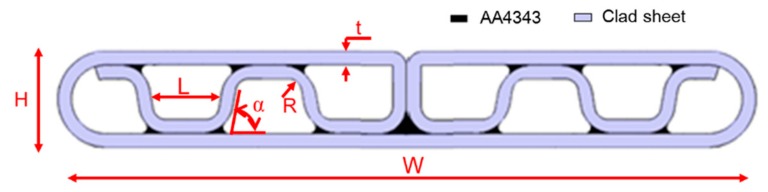
Dimensional parameter definition and material distribution of the folded MPFT with 8 ports.

In this study, typical folded MPFTs with eight ports were prepared. The material and dimensions of the MPFT are shown in Figure 2, where *W*, *H*, *L*, α, *R* and *t* are the tube width, tube height, port width, folding angle, folding radius and wall thickness, respectively. The geometrical parameters chosen in current work were typical for a folded MPFT (Table 1). The folded MPFT was made of an AA4343/AA3003/AA4343 sandwich sheet by using a roll forming process. After folding, the process of brazing melted the solder (AA4343) to fill the folding gaps and made the folded MPFT airtight.

**Figure 3:**
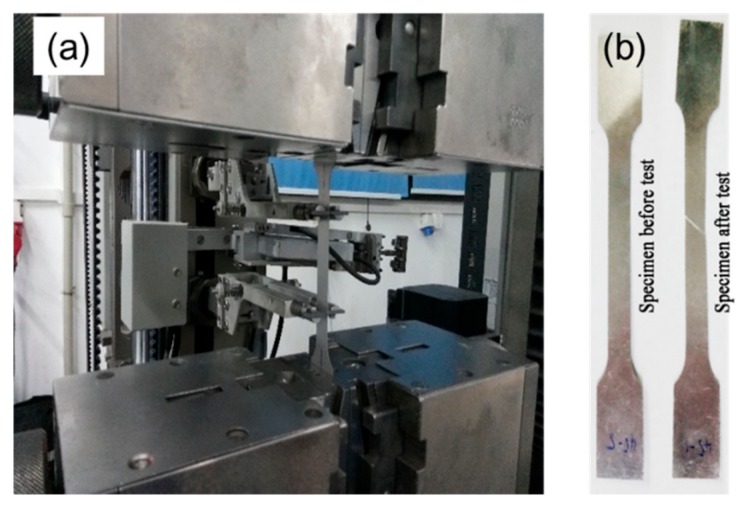
Tensile test of the materials: (**a**) equipment, and (**b**) specimens for tensile tests.

To get the properties, tensile tests for both the clad sheet and solder were carried out with flat dog-bone specimens (Figure 3). The thickness and gauge length of the specimen are 0.23 mm and 50 mm, respectively. The strain was measured by a mechanical extensometer (BTC-T1-FR020, ZwickRoell Co. Ltd., Ulm, Germany) with a strain rate of 10^−4^/s. At least two specimens were used in each experiment to ensure the repeatability of the results.

### 2.2. In-Plane Bending

**Figure 4:**
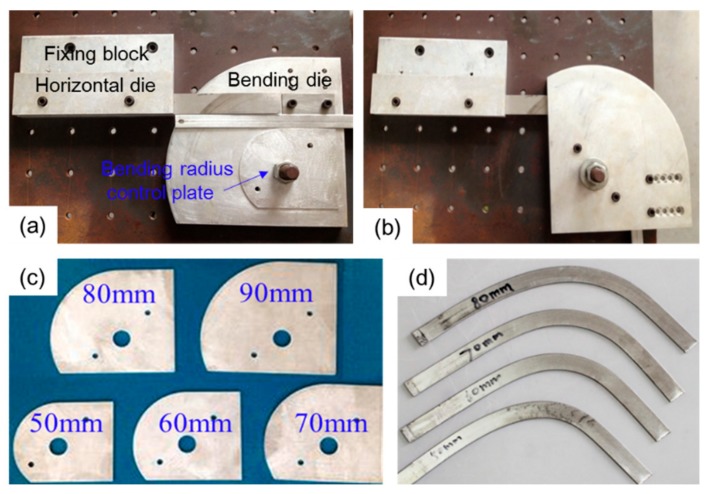
In-plane bending test: (**a**) bending device at initial position; (**b**) bending position at 90°, (**c**) bending radius control plate and, (**d**) bent specimens with different bending radius.

In order to evaluate the influence of in-plane bending on the properties of folded MPFTs, in-plane bending tests were carried out (Figure 4). A typical rotary draw bending device, which consisted of a horizontal mold, fixing block, bending radius control plate and bending die, was manufactured (Figure 4a). One end of the tube was fixed within the bending die and the other end was inserted into the horizontal mold. As the bending die rotated, the tube was bent within the plane associated with the flat surface of the folded MPFT (Figure 4b). Through changing the bending radius control plate (Figure 4c), different bent samples were prepared (Figure 4d).

### 2.3. Pressure Bearing Capacity Test

**Figure 5:**
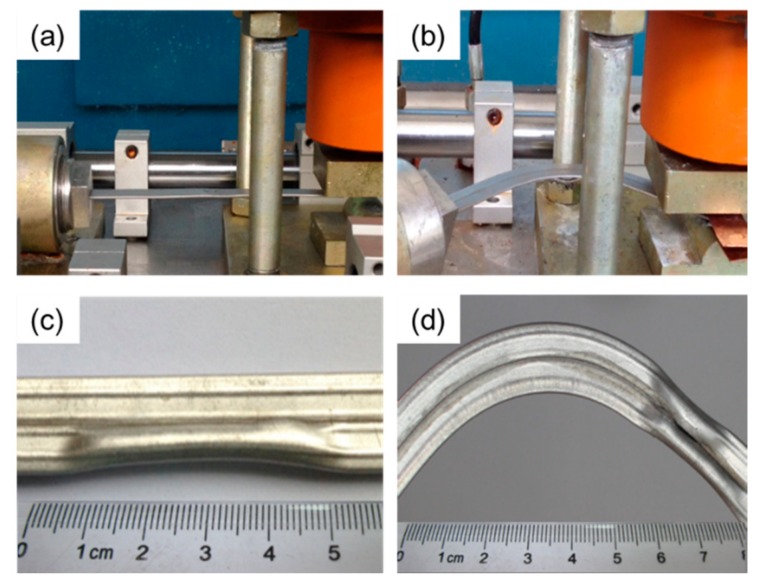
Pressure bearing test: (**a**) test device for straight specimen, (**b**) test device for bent specimen, (**c**) straight specimen after failure and, (**d**) bent specimens after failure.

As pressure bearing capacity is crucial for the application of the folded tube, pressure bearing tests were conducted for both the straight and bent MPFT specimens. Figure 5a,b shows the the straight specimen and the bent specimen. A detailed description of the testing procedures can be found in the work of Tang et al. [13]. In this study, the device was adjusted to be able to test the pressure bearing capacity of bent specimens (Figure 5b). The burst pressure and the bulging degree were recorded.

### 2.4. FE Modeling

**Figure 6:**
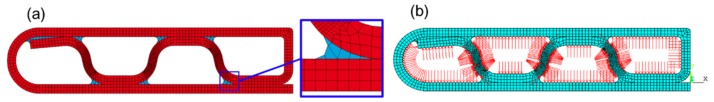
FE simulation of pressure bearing test: (**a**) geometry of the MPFT section, (**b**) boundary condition of the pressure bearing test.

The pressure bearing test of straight folded MPFT was modeled by the commercial software ANSYS (ANSYS Inc., Downingtown, PA, USA). Dynamic/Explicit solver was used in this study. Figure 6 presents the FE model and applied boundary conditions of the burst pressure test. The wall material (AA4343/AA3003/AA4343 clad material) and solder material (AA4343) were treated as different materials, as shown in Table 2. The ribs between the ports, which were the weakest part of the structure, were fine-meshed with four-node bilinear quadrilateral integration elements (Figure 6a).

Figure 6b shows the pressure loading condition. The pressure was applied on the inner surface of the tube and increased linearly up to failure in simulation. Only half of the folded MPFTs were modeled due to symmetry. Symmetry boundary condition was applied on the right-hand side wall. Plane strain condition was assumed considering that the length of a folded MPFT (>300 mm) was much longer than the dimensions of the cross section (16 mm × 1.8 mm).

**Figure 7:**
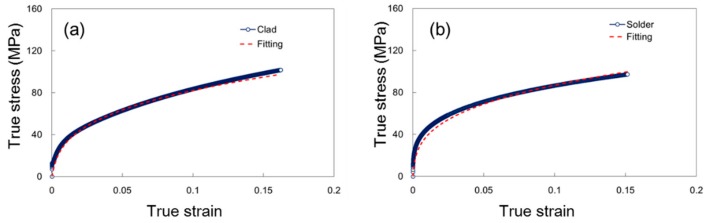
Uniaxial stress–strain curves of (**a**) clad specimen and (**b**) AA4343 specimen.

The classic J2 plasticity theory was employed for the simulations. The relation between equivalent strain ε and the von-Mises stress, σ, of both materials followed the Ramberg-Osgood equation [14]

(1) $$\varepsilon \;=\;\frac{\sigma }{E}+{\left(\frac{\sigma }{K}\right)}^{\frac{1}{n}}$$

where  E is Young’s modulus and *K* and *n* are parameters associated with the Ramberg-Osgood model. The parameters listed in Table 2 were obtained from fitting the experimental tensile stress–strain curves of two materials (Figure 7). These parameters were used for all subsequent FE simulations. In addition, a failure criterion based on the critical equivalent strain was employed to determine if the MPFT under investigation failed or not. The critical strain of the two materials, determined from the tensile tests, are listed in Table 2.

## 3. Results and Discussion

### 3.1. Comparison between Experimental and Simulated Results

**Figure 8:**
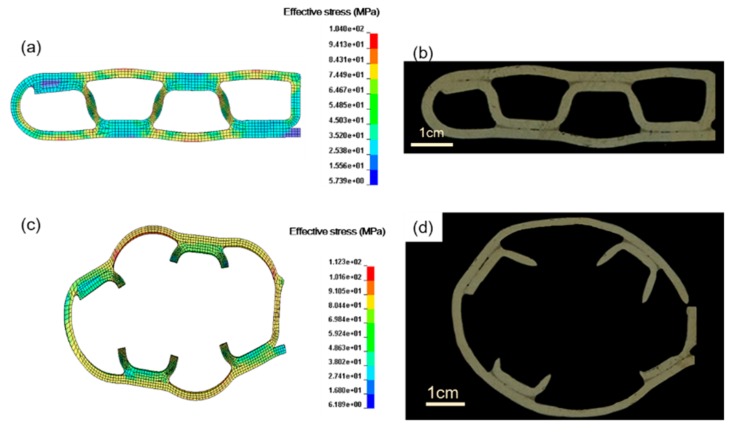
Comparison of the experimental and predicted deformed configurations at (**a**,**b**) certain inner pressures (17.5 MPa for experiment and 18 MPa for simulation) and (**c**,**d**) ultimate failure.

Figure 8 compares the deformed configurations of the MPFTs at a certain inner pressure (18 MPa for FE simulations and ~17.5 MPa for experiments) and the ultimate failure between the experiment and simulation. The simulation results show that, by increasing the inner pressure, the upper and lower walls (single layer regions) bulged gradually (Figure 8a). A good match was obtained between the predicted and experimental results (Figure 8).

To describe the bulging of the upper and lower walls, the bulging ratio was defined with

(2) $$\alpha \;=\;\frac{H_{1}-H_{0}}{H_{0}}$$

where $H_{0}$ and $H_{1}$ are the initial and deformed heights under inner pressure [13]. The measured and predicted bulging ratios agree well with each other, with only a 4.5% error at the pressure close to ultimate failure. It is worth noting that the service pressure of the tube is around 6 MPa, where the corresponding bulging ratios obtained from both experiments and simulations are less than 5% (Table 3).

Figure 8c,d shows the failure mode of the folded tube under ultimate inner pressure. The simulation result agrees well with the experiment: four joints between the outer wall and the inner ribs keep intact, while the three inner ribs break. There is still some small difference on breaking position on ribs: for the experiments, the ribs break at the end close to the joint, while for the FE simulation, the ribs break in the middle. This difference may be caused by thinning of the ribs in the folding process.

The inner ribs become thinner, necking and eventually cracking under inner pressure. Ultimate failure pressure can be obtained with FE simulation. Figure 8c shows the deformed configuration of the tube section after the fracture of the inner ribs (at inner pressure of 18.26 MPa). These simulations are confirmed by the corresponding experimental results with the relative error less than 5% (Figure 8b,d and Table 3), where the relative error was calculated as the ratio of the absolute difference (between simulation and averaged experiment result) to the experiment result. The failure pressures are very close to the pressures where the bulging ratios were measured, which indicates that the bulging ratio could be a good indicator to evaluate the status of a folded tube under service. The folded MPFT investigated in current work is bound to fail if its bulging ratio reaches 23%. The bulging ratio can be easily observed from the outer appearance of an MPFT, while thinning, necking and cracking of inner ribs can be hardly detected.

### 3.2. Optimization on the Section of Folded MPFT

**Figure 9:**
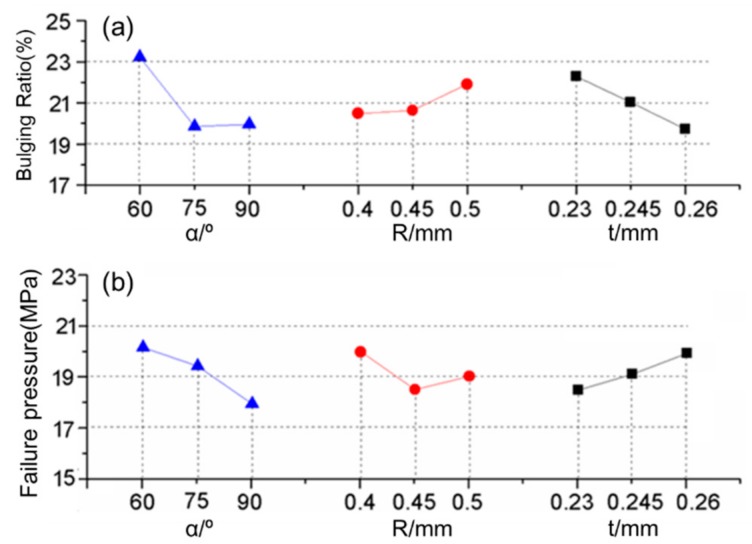
Predicted (**a**) bulging ratio and (**b**) failure pressure as a function of the folding angle, folding radius and wall thickness.

Optimization on the pressure bearing capacity of folded MPFTs is of great significance. According to the above FE analysis, failure of the tube closely relates to the breaking of inner ribs under inner pressure. In order to strengthen the inner ribs, folding angle  α, folding radius *R* and wall thickness *t* are the parameters to be optimized in this study. Therefore, experiments and simulations, designed orthogonally (Table 4), were conducted to figure out the most influential parameters associated with the performance of a folded MPFT. Figure 9 presents the effects of the three parameters on the bulging ratio at a pressure of 17.5 MPa, as well as the failure pressure. The bulging ratio decreased with increasing the folding angle and wall thickness, and decreased with increasing the folding radius. The failure pressure decreased with an increase of the bending angle and folding radius, and decrease of the wall thickness.

Among the combinations, the case of α = 75°, *R* = 0.4 and *t* = 0.26 exhibited the best on both the bulging ratio and failure pressure. A corresponding FE simulation was carried out to verify this case and the simulation results are listed in Table 5. Compared to the initial design, the bulging ratio decreased from 23.3% to 19.8% and the failure pressure increased from 18.28 MPa to 20.9 MPa.

### 3.3. Pressure Bearing Capability of Bent MPFTs

**Figure 10:**
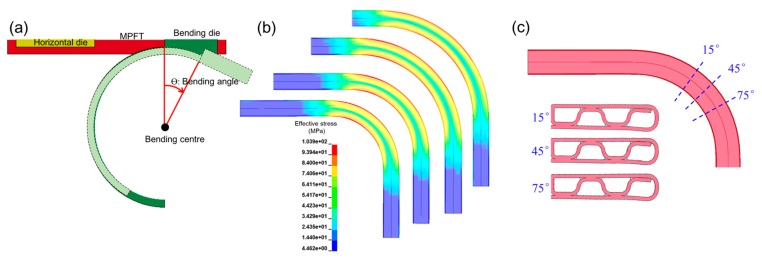
FE simulations of burst pressure tests: (**a**) FE model, (**b**) von Mises stress with different bending radius, (**c**) details associated with wall thinning.

FE simulations on the in-plane bending of folded MPFTs were carried out to investigate the relevant influence (Figure 10). During the bending process, the bending angle increased gradually from 0° to 90° (Figure 10a). It can be seen that after bending, the minimum stress is located at the central line of the tube cross section, while the maximum stress appears at the outer wall for all the bending radii studied (Figure 10b). Figure 10c shows the deformed cross section of the bent MPFTs at bending angle of 15°, 45° and 75°. During bending, the further the outer wall is from the bending center, or the larger the bending angle is, the more the wall thins.

For quantitative analysis of the in-plane bending effect, the maximum von Mises stress and wall thinning ratio of specimens with different bending radius were extracted from the FE simulations. The thinning ratio of the outer wall is defined as

(3) $$\Delta \;=\;\frac{t_{1}-t_{0}}{t_{0}}$$

where $t_{0}$ and $t_{1}$ are the initial and deformed thickness of the tube outer wall (Figure 10c) [11].

**Figure 11:**
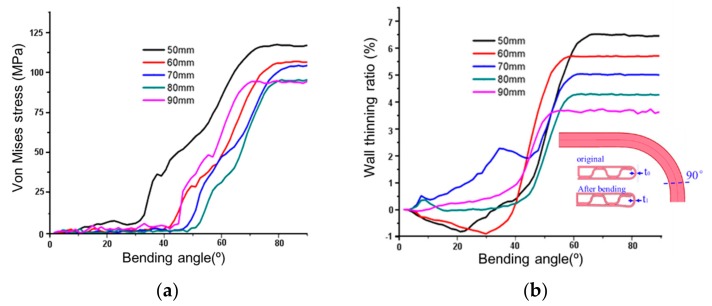
FE simulation on the in-plane bending of folded MPFTs: (**a**) von Mises stress and (**b**) Wall thinning ratio as a function of the bending angle.

Figure 11a shows that the maximum von Mises stress increases monotonically along with bending (bending angle increases from 0 to 90°). Apparently, at a bending angle smaller than ~30°, the cooperation of the rotary bending radius plate and the horizontal mold mainly pulled the sample horizontally, and this did not lead to significant deformation. The stress induced was also low. At bending angles between ~30° to 75°, a significant increase in stress was obtained, where bending deformation mainly occurred. The bending process reached its maximum around the bending angle of ~75°. Therefore, maximum stress kept constant beyond the bending angle of ~75°. Similarly, three regions were observed for the wall-thinning ratio. At low bending angle, slight wall thickening was observed for the sample with bending radii of 50 mm and 60 mm, while slight wall thinning for 70 mm, 80 mm and 90 mm. When increasing the bending angle up to ~60°, wall thinning became more significant. Beyond that angle, the wall-thinning ratio was large (as large as 7%) but was constant.

Table 6 lists experimental failure pressure with bending radius varying from 50 mm to ∞ (i.e., straight tube). The pressure bearing capability was not reduced obviously after bending with different bending radius. The average failure pressure is around 16.8 MPa, which is 6.5% smaller than the failure pressure of the straight tube. This demonstrates that, after bending, the folded MPFTs can still retain most of the pressure bearing capability.

## 4. Conclusions

In this paper, the pressure bearing capability of folded MPFTs was investigated with finite element analysis and a burst pressure test. Optimization on the design of the folded MPFTs was discussed. The effect of in-plane bending on the property of the folded MPFTs was also investigated. Based on the results, the main conclusions can be drawn as follows:

(1) The folded MPFT’s failure is mainly caused by breaking of the inner ribs. The bulging ratio is low (<5%) under the service inner pressure. However, this ratio increases to ~23% before burst failure. Therefore, the bulging ratio is a good indicator to tell whether the fold MPFT is about to fail or not.
(2) An improved design of the folded MPFTs was proposed based on the orthogonal FE simulations. The improved design can increase pressure bearing capability from 18.28 MPa to 20.9 MPa and decrease the bulging ratio from 23.3% to 19.8%.
(3) The bending process will lead to a 6.5% thinning rate in the tube’s outer layers. The pressure bearing capability has not been significantly weakened after bending with different bending radius. It is therefore revealed that the folded MPFTs retain most of the pressure bearing capability after in-plane bending.

Compared to the folded MPFT, the extruded MPFTs are more often employed in industry. Pressure bearing properties of extruded MPFTs have been extensively investigated. The processes of seam welding, extrusion, rolling and brazing affect both the microstructure and the mechanical performance [7,15]. In comparison, the influencing factors on the properties of folded MPFTs were rarely investigated [16,17]. The current work provides valuable information on the design of folded MPFTs. Of course, the design of an MPFT is to satisfy, not only the pressure bearing capability, but also the heat transferring, corrosion resistance, etc. These aspects associated with MPFTs fabricated in terms of folding will be good topics to be investigated in the future.

## Figures and Tables

**Table 1:** Dimensional parameters of the folded MPFT.

*W*/mm	*H*/mm	*L*/mm	*α*/°	*R*/mm	*t*/mm
16	1.8	1.6	72	0.50	0.23

**Table 2:** Material parameters used in the FE simulations.

Material	*E*/GPa	*K*/MPa	*n*	Critical Strain
Clad sheet	5.40	183.60	0.32	0.162
Solder Material	15.26	165.35	0.28	0.151

**Table 3:** Experimental and simulated bulging ratio and failure pressure.

-	Experiment				Simulation	Relative Error
	Test 1	Test 2	Test 3	Average		
Bulging ratio at service pressure	4.06	4.46	3.84	4.12	4.33	5.1%
Bulging ratio at pressure close to failure	21.36	23.20	22.35	22.30	23.3	4.5%
Failure pressure/MPa	17.70	18.20	18.00	17.97	18.28	1.7%

**Table 4:** Orthogonal experiment among the parameters under optimization.

α/°	*R*/mm	*t*/mm
60, 75, 90	0.4, 0.45, 0.5	0.23, 0.245, 0.26

**Table 5:** Comparison between the original and optimized MPFTs.

Factors	*α*/°	*t*/mm	*R*/mm	Failure Pressure/MPa	Bulging Ratio at 17.5 MPa
Original	72	0.23	0.5	18.28	23.3%
Optimized	75	0.26	0.4	20.9	19.8%

**Table 6:** Failure pressure of MPFT with different radius from experiments.

Bending Radius	50 mm	60 mm	70 mm	80 mm	90 mm	∞ (Straight)
Failure pressure (Exp.)	16.1 MPa	16.6 MPa	17.1 MPa	17.5 MPa	17.8 MPa	17.97 MPa
